# Risk of venous thromboembolism in hospitalised cancer patients in England—a cohort study

**DOI:** 10.1186/s13045-016-0291-0

**Published:** 2016-07-26

**Authors:** Sonia Ratib, Alex J. Walker, Tim R. Card, Matthew J. Grainge

**Affiliations:** 1 Centre of Evidence Based Dermatology, King’s Meadow Campus, Lenton Lane, University of Nottingham, Nottingham, NG7 2RN UK; 2 School of Life Sciences, Queens Medical Centre, A Floor, West Block, Nottingham, NG7 2UH UK; 3 Division of Epidemiology and Public Health, School of Medicine, University of Nottingham, Clinical Sciences Building 2, City Hospital, Nottingham, NG5 1PB UK

**Keywords:** Cancer, Venous thrombosis, Hospitalisation, Epidemiology, Chemotherapy

## Abstract

**Background:**

Venous thromboembolism (VTE) is a well-recognised and life-threatening complication in patients with cancer. However, the precise risk of VTE in hospitalised cancer patients in England has not been previously reported.

**Methods:**

We conducted a cohort study using linked Hospital Episodes Statistics and Office for National Statistics mortality data. We determined the risk of VTE separately for 24 cancer sites following first hospitalisation for cancer (index date) and how this varied by age, proximity from hospital admission, administration of chemotherapy and calendar time.

**Results:**

Between 1998 and 2012, 3,558,660 patients were hospitalised for cancer. The cancer sites with the highest risk of VTE during initial hospitalisation for cancer were pancreatic (4.9 %), ovarian (4 %) and liver (3.8 %). The three cancer sites with the highest risk of first VTE event within 6 months from discharge were pancreatic (3.7 %), oesophagus (3 %) and stomach (2.8 %). For most cancers, the risk of VTE within 6 months from discharge was higher amongst patients who underwent chemotherapy compared to those who did not. The impact of age on risk of VTE varied considerably between cancer sites.

**Conclusions:**

The risk of VTE amongst patients hospitalised for cancer varies greatly by cancer site, age, proximity from hospital admission, and chemotherapy administration.

**Electronic supplementary material:**

The online version of this article (doi:10.1186/s13045-016-0291-0) contains supplementary material, which is available to authorized users.

## Background

Venous thromboembolism (VTE) is responsible for approximately 25,000 deaths each year in the UK, and it is well established that patients with cancer are at higher risk of VTE compared to the general population [1–3]. The estimated annual incidence of VTE in the cancer population is 1.3 %, and the risk of death is higher for cancer patients with VTE than for those without [4–8]. Given the ageing population and increased early diagnosis, more people are living with cancer in the UK than ever before [9]. Therefore, the prevention of a potentially fatal cancer-associated VTE amongst patients is of paramount importance. Furthermore, the long-term consequences of VTE such as post-thrombotic syndrome (PTS) are more of an issue now as people with cancer are living long enough now to develop them. The cost to the NHS for management of PTS is significant and covered in the Department of Health enquiring into VTE [1].

Current UK guidelines, published by the National Institute for Health and Care Excellence (NICE), recommend prophylaxis for VTE for cancer patients admitted to hospital, but only routinely to those hospitalised for 3 or more days or who are expected to have ongoing reduced mobility [10]. Prophylaxis is cheap and highly effective (around 50 to 70 % reduction) [11, 12]; however, to prevent unnecessary harm from thromboprophylaxis and its associated adverse effects, careful consideration must be given to identify patients who are most at risk so that prophylaxis can be appropriately targeted.

Previous studies and a recent report from the Centers for Disease Control and Prevention confirm hospitalisation is an important risk factor for VTE and emphasise the need for greater awareness of VTE risks and implementation of preventative measures in hospital [13, 14]. To date, a limited number of hospital-based studies in patients with cancer (outside of the UK only) have been conducted, and the majority of studies did not determine the risk of VTE following discharge [15–18]. Of the two that did, one study was limited to a select patient group and the other did not determine how the risk of re-admission for VTE varies by potential risk factors [15, 16].

There is therefore a need for a hospital-based cohort study in patients with cancer in England to determine contemporary and precise estimates of the risk of VTE, taking into account risk factors such as age, cancer site, proximity from admission and chemotherapy administration. Such risk stratification could be used to inform future clinical guidelines and optimise the use of prophylactic anticoagulation when patients are admitted to hospital with cancer.

This study uses the English Hospital Episode Statistics (HES) and linked Office for National Statistics (ONS) death certificate data to determine the risk of VTE in hospitalised cancer patients, during admission and post-discharge, and stratified by risk factors.

## Methods

### Data source and patients

We conducted a cohort study using the Hospital Episode Statistics (HES) database, which contains details on all inpatient (except accident and emergency) and day case admissions to English NHS hospitals from 1989. More than 12 million admission records are added each year [19]. The database is managed by the Health and Social Care Information Centre and contains data on hospitalisations, which are broken down into periods of care seen by consultants (episodes). The primary diagnosis (the main reason the patient is receiving care) per episode is indicated along with all secondary diagnoses (any relevant comorbidities and illnesses acquired whilst in hospital). A diagnosis is coded using the ICD-10 (International Classification of Diseases, 10th revision), and all procedures which take place during the admission are coded using the OPCS-4 (Office of Population, Censuses and Surveys’ classification of surgical operations and procedures, fourth revision). HES is linked to the ONS death registry which provides the date of death for all deceased patients.

We selected patients who had a first cancer diagnosis recorded in HES (ICD-10 Chapter II, C00-C97, excluding non-melanoma skin cancer) between 1 January 1998 and 31 October 2012, as this was the period the HES data were available for at the time of writing. Patients who had a VTE event were identified. Patients were excluded if:

- Under 18 years of age at first cancer diagnosis
- Had a VTE diagnosis in a hospital admission prior to the cancer admission

Data were analysed separately for the 24 most common cancer sites (based on 2007 UK incidence data). Cancer sites not included within these were categorised as ‘Other’. ‘Unknown primary’ site consisted of metastatic cancers with no known primary cancer site (C77–C80). Cancer site classification was based on the first occurring cancer, and the corresponding date was assumed to be the date of diagnosis (termed index date from this point onwards). Ethical approval was given by the ONS for this study (reference number RU863/NIC-165667-FH1W1).

### VTE event

For the cancer patients, a VTE diagnosis was defined as (i) having a hospital admission for pulmonary embolism (ICD-10, I26) or venous thrombosis (ICD-10, I80, I81 or I82), (ii) a diagnosis with one of the above codes during a hospital admission for another reason and (iii) having one of the above codes as underlying cause of death. The first VTE event concurrent with or following the index date was selected as the outcome of interest.

### Chemotherapy

Patients receiving inpatient therapy were identified using OPCS-4 codes for chemotherapy (X72.1, X72.2, X72.3 and X73.1).

### Statistical methods

The risk of VTE was stratified by timing of the first VTE event, that is, whether the event occurred during the same hospitalisation as the index date or as re-admission in the 6 months following discharge, for all 24 cancers sites. Further stratification by age-group (<60, 60–80 and >80 years) was performed for the four most commonly diagnosed cancers in the UK (breast, lung, bowel and prostate), those found to be at high risk of VTE (according to our data) and all cancers combined. The relative risk of first VTE as a re-admission within 6 months from discharge amongst those who had a record of chemotherapy compared to those who did not was determined using logistic regression, for all cancer sites.

Trends in VTE risk over time (assigning patients to year of index date) were investigated for the four most commonly diagnosed cancers. Patients whose first VTE event was concurrent with their index date were removed from this analysis to ensure the VTE event was subsequent to the cancer diagnosis. Patients whose index date was in 2012 were also excluded from this part of the analysis as data were not available for the full calendar year. To control for differing length of hospital stay (a marker of cancer severity), we repeated the analysis stratified by short-term (<3 days) and prolonged stay (≥3 days). This cutoff was chosen according to NICE VTE guidelines (NICE guidelines, 2010). We also conducted a sensitivity analysis to determine if trends for the whole cohort were different to the subgroup of patients whose primary diagnosis was cancer.

In addition to risk, absolute rates of VTE were determined to account for varying length of survival by type of cancer. The rates were presented by cancer site and timing of VTE event in relation to hospitalisation: during hospitalisation or 6 months post-discharge. Person-time at risk commenced at the time of index date or time from discharge for each respective group. Patients were followed up until they developed a VTE event, died, 6 months post-discharge, or 31 October 2012 (last data collection date), whichever was earliest. Rates were calculated as the number of first VTE events divided by person-time (per 1000 person-years). VTE events concurrent with start of follow-up were excluded (as these patients did not contribute person-time years). All data management and statistical analysis were performed using Stata 12 (Statacorp, 4905 Lakeway Drive, College Station, Texas 77845, USA).

## Results

### Patients

A total of 3,558,680 patients were identified with a hospital admission for cancer between 1998 and 2012. The median age at index date was 70 (IQR 59.6, 78.7) years. Of these patients, 108,770 (3.06 %) had a VTE anytime between index date and up to 6 months from discharge; just under two-thirds of these (*n* = 66,954; 61.6 %) had their first VTE during the hospitalisation for cancer (Table 1). Of the 155,650 patients who had a VTE any time during the study period, *n* = 70, 725 (45.4 %) had a PE and *n* = 84,925 (54.6 %) had a DVT as their first VTE event. There were 6235 (4 %) patients who died from their VTE during the study period and the median (interquartile range) follow-up time was 1.6 (0.31, 4.40) years.

**Table 1:** Patient characteristics

Characteristic	No. of patients^a^	%
Sex		
Male	1,803,145	50.7
Female	1,755,535	49.3
Age at first cancer diagnosis (years)		
18–40	154,617	4.3
41–60	761,059	21.4
61–80	1,879,373	52.8
>80	763,631	21.5
Mean (SD)	68.2 (14.3)	
Median (IQR)	70 (59.6, 78.7)	
Follow-up time (years)		
Total	12,028,985	
Median (IQR)	1.70 (0.33, 5.46)	
First VTE event		
During hospitalisation	66,954	43.02^b^
Within 6 months following discharge	41,816	26.87^b^
Beyond 6 months following discharge	46,880	30.12^b^
Entire study	155,650	

*SD* standard deviation, *IQR* interquartile range

^a^Unless otherwise stated

^b^Total number of patients who had a VTE during the entire study

### Risk of first VTE by cancer site and timing from index date

For the majority of cancers, the risk of VTE during hospitalisation was higher than in the first 6 months post-discharge (1.88 vs. 1.42 % respectively, overall) (Table 2). The cancer sites with the highest proportion of VTE events during initial hospitalisation for cancer were pancreatic (4.89 %), ovarian (4.01 %) and liver (3.84 %). In contrast, VTE occurred in less than 0.5 % of patients with malignant melanoma, oral and laryngeal cancer. Of the 2,943,792 patients alive at discharge and without a prior VTE event, the three cancer sites with the highest risk of a VTE within 6 months were pancreatic (3.66 %), oesophagus (2.98 %) and stomach (2.84 %).

**Table 2:** First VTE event (%) stratified by cancer site and timing of event, up to 6 months from discharge

First VTE event	During hospitalisation			Within 6 months following discharge			Total		
Cancer site	No. of people	No. with VTE	%	No. of people alive at discharge & no previous VTE	No. with VTE	%		No. with VTE	%
Breast	525,053	4843	0.92	485,009	3643	0.75	525,053	8486	1.62
Lung	395,671	9808	2.48	278,182	6436	2.31	395,671	16,244	4.11
Bowel	432,308	7369	1.70	364,489	5635	1.55	432,308	13,004	3.01
Prostate	384,078	5876	1.53	335,231	2191	0.65	384,078	8067	2.10
Non-Hodgkin lymphoma	134,096	2979	2.22	113,989	2155	1.89	134,096	5134	3.83
Malignant melanoma	86,496	318	0.37	82,445	155	0.19	86,496	473	0.55
Bladder	241,152	2057	0.85	217,217	1547	0.71	241,152	3604	1.49
Kidney	73,273	2229	3.04	60,755	724	1.19	73,273	2953	4.03
Oesophageal	98,668	1395	1.41	79,812	2379	2.98	98,668	3774	3.82
Stomach	86,454	2044	2.36	66,314	1886	2.84	86,454	3930	4.55
Pancreatic	78,579	3846	4.89	52,296	1915	3.66	78,579	5761	7.33
Leukaemia	108,405	1913	1.76	86,648	928	1.07	108,405	2841	2.62
Uterus	74,346	1113	1.50	68,516	705	1.03	74,346	1818	2.45
Ovarian	70,613	2834	4.01	57,162	1314	2.30	70,613	4148	5.87
Oral	69,827	301	0.43	62,784	378	0.60	69,827	679	0.97
Brain	69,362	1545	2.23	55,107	1353	2.46	69,362	2898	4.18
Multiple myeloma	59,610	1058	1.77	48,944	1033	2.11	59,610	2091	3.51
Liver	52,242	2005	3.84	36,485	783	2.15	52,242	2788	5.34
Cervix	33,618	530	1.58	30,596	459	1.50	33,618	989	2.94
Laryngeal	25,918	127	0.49	22,879	86	0.38	25,918	213	0.82
Testicular	22,985	172	0.75	22,481	201	0.89	22,985	373	1.62
Bone/connective tissue	30,023	483	1.61	26,490	293	1.11	30,023	776	2.58
Thyroid	22,718	122	0.54	21,368	56	0.26	22,718	178	0.78
Mesothelioma	22,354	361	1.61	17,350	349	2.01	22,354	710	3.18
Other site	102,771	1792	1.74	87,853	1190	1.35	102,771	2982	2.90
Unknown	258,040	9834	3.81	163,390	4022	2.46	258,040	13,856	5.37
Total	3,558,660	66,954	1.88	2,943,792	41,816	1.42	3,558,660	108,770	3.06

### Risk of VTE by age and timing from index date

For all cancers combined, the risk increased from 1.4 % in those less than 60 years to 2.3 % in those over 80 years (Table 3). However, for the cancers we considered with a poor prognosis (lung, liver and pancreatic), the risk of VTE during hospitalisation decreased with age.

**Table 3:** First VTE event (%) during hospitalisation stratified by cancer site and age-group

	Age (years)								
Cancer site	<60			60–80			>80		
During hospitalisation	No. of people	No. with VTE	%	No. of people	No. with VTE	%	No. of people	No. with VTE	%
Breast	231,286	1166	0.50	220,311	2112	0.96	73,456	1565	2.13
Lung	60,594	1820	3.00	249,044	5775	2.32	86,033	2213	2.57
Bowel	76,305	984	1.29	247,306	4068	1.64	108,697	2317	2.13
Prostate	32,460	255	0.79	239,913	3516	1.47	111,705	2105	1.88
Ovarian	25,834	740	2.86	34,245	1527	4.46	10,534	567	5.38
Pancreatic	13,278	700	5.27	44,386	2259	5.09	20,915	887	4.24
Liver	11,020	514	4.66	28,717	1101	3.83	12,505	390	3.12
All cancers	915,676	12,787	1.40	1,879,373	36,692	1.95	763,631	17,475	2.29

### Trends of VTE by calendar year

**Fig. 1:**
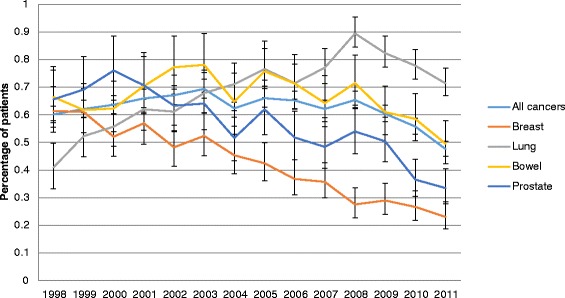
Percentage of patients with first VTE during hospitalisation by year of cancer diagnosis

**Fig. 2:**
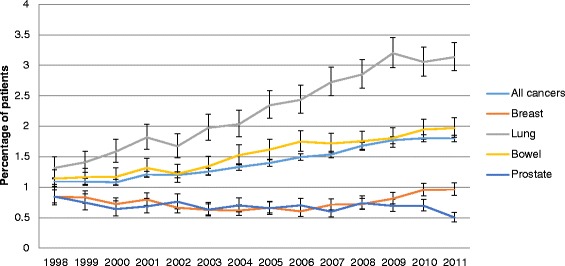
Percentage of patients with first VTE within 6 months following discharge by year of cancer diagnosis

Figures 1 and 2 display the risk of VTE during hospitalisation and within 6 months of discharge, respectively, by year of index date. With respect to the risk during hospitalisation, the trends varied by cancer site. Overall, the risk decreased with time, especially for breast and prostate cancer. In contrast, for lung cancer, the risk of VTE increased between 1998 and 2008. With respect to the risk of VTE as a re-admission, there was an overall increase over the calendar period. The increase was relatively small for breast and prostate but significant for lung and bowel, increasing twofold for lung and just over 50 % for bowel from 1998 to 2011.

### Trends of VTE by calendar year stratified by length of stay

When stratified by hospital duration, the reduction of VTE over time is less pronounced for breast cancer but the rise amongst lung cancer patients still remains (Additional file 1: Figure S1). Trends by length of stay were similar between the overall cohort (Additional file 1: Figure S1) and the subgroup of patients whose primary diagnosis was cancer (Additional file 2: Figure S2).

### Risk of VTE by chemotherapy

Of the study population, a total of 719,257 patients (20.2 %) received inpatient chemotherapy during the study period and the median time from index date to chemotherapy was 46 (IQR 16, 104) days. The number of people who received chemotherapy during their initial hospitalisation was 250,638 (7 %). Of those who were discharged without a VTE, and followed-up for up to 6 months, 22.7 % received chemotherapy (Table 4). For these patients, the odds ratio of VTE in those who underwent chemotherapy compared to those who did not was 1.75 (95 % CI 1.72, 1.79). The cancer sites associated with the highest risk of VTE within 6 months from discharge, if chemotherapy was undertaken, were pancreatic (5.2 %), stomach (4.87 %) and oesophageal (4.67 %). The cancer sites with the highest risk of VTE amongst patients not receiving chemotherapy were pancreatic (3.20 %), brain (2.52 %) and ovarian (2.43 %). For all cancer sites, except brain, ovarian, multiple myeloma and oral cancer, the proportion of chemotherapy patients who had a VTE event was statistically significantly higher than those who did not undergo treatment (*p* < 0.05 for all instances).

**Table 4:** First VTE event within 6 months from discharge (%) stratified by cancer site and chemotherapy

Cancer site	No. of people alive and no VTE during hospitalisation	Chemotherapy	VTE	%	No chemotherapy	VTE	%
Breast	485,009	138,776	1871	1.35	346,233	1772	0.51
Lung	278,182	71,155	2389	3.36	207,027	4047	1.95
Bowel	364,489	96,577	1853	1.92	267,912	3782	1.41
Prostate	335,231	17,687	180	1.02	317,544	2011	0.63
Non-Hodgkin lymphoma	113,989	60,495	1165	1.93	53,494	990	1.85
Malignant melanoma	82,445	4088	20	0.49	78,357	135	0.17
Bladder	217,217	32,418	291	0.90	184,799	1256	0.68
Kidney	60,755	4458	61	1.37	56,297	663	1.18
Oesophageal	79,812	22,786	1063	4.67	57,026	1316	2.31
Stomach	66,314	14,916	726	4.87	51,398	1160	2.26
Pancreatic	52,296	12,015	625	5.20	40,281	1290	3.20
Leukaemia	86,648	23,957	287	1.20	62,691	641	1.02
Uterus	68,516	7615	146	1.92	60,901	559	0.92
Ovarian	57,162	28,259	613	2.17	28,903	701	2.43
Oral	62,784	12,080	114	0.94	50,704	264	0.52
Brain	55,107	6726	135	2.01	48,381	1218	2.52
Multiple myeloma	48,944	18,175	348	1.91	30,769	685	2.23
Liver	36,485	5228	155	2.96	31,257	628	2.01
Cervix	30,596	7651	177	2.31	22,945	282	1.23
Laryngeal	22,879	2711	15	0.55	20,168	71	0.35
Testicular	22,481	9119	103	1.13	13,362	98	0.73
Bone/connective tissue	26,490	4231	78	1.84	22,259	215	0.97
Thyroid	21,368	717	3	0.42	20,651	53	0.26
Mesothelioma	17,350	4908	120	2.44	12,442	229	1.84
Other site	87,853	36,920	476	1.30	50,933	714	1.40
Unknown	163,390	25,095	1128	4.49	138,295	2894	2.09
Total	2,943,792	668,763	14,142	2.11	2,275,029	27,674	1.22

### VTE rates by cancer site and timing from index date

For all cancer sites, the absolute rate of VTE was higher during hospitalisation compared with rates in the first 6 months following discharge. In the first 6 months following discharge, the cancer sites associated with the highest rates were pancreatic (11.9 per 1000 person-years; CI 11.3–12.4), oesophageal (7.8; CI 7.4–8.1) and lung (6.8; CI 6.7–7.0). The overall rate of VTE was 3.34 per 1000 person-years (95 % CI 3.31–3.37). (Additional file 3: Figure S3).

## Discussion

### Main findings

We found that more people developed VTE in their initial hospitalisation than in the subsequent 6 months, for most cancer types. Regardless of how we assessed VTE, pancreatic cancer was associated with the highest risk of VTE of all measured cancer types, both overall and specifically amongst those who underwent chemotherapy. The overall risk of VTE in people hospitalised for cancer was 3.06 % and overall varied from 1.88 % during hospitalisation to 1.42 % within 6 months from discharge; in those with pancreatic cancer, the equivalent risk was 4.89 and 3.66 %, respectively. For cancer types with a poor prognosis (e.g., lung), there was a negative association between age and risk of VTE. For most cancer types, the risk of VTE within 6 months from discharge was higher amongst those who received chemotherapy than those who did not. Compared with previous work, we found important differences in time trends depending on whether VTE was assessed during the initial hospitalisation or in the ensuing 6 months. In particular, re-admission rates for VTE from 1998 to 2011, increased by twofold in patients with lung cancer and 50 % in those with bowel cancer.

### Strengths and limitations

This is the first study to describe the risk of VTE in a hospitalised cancer population in the UK and is one of the largest studies worldwide on this topic. The large sample size gives precise risk estimates stratified by cancer type, including those of lower prevalence. As the HES database incorporates all inpatient and day case hospital admissions taking place in England, our results are nationally generalisable. Moreover, we have been able to distinguish VTE events which were recorded during the cancer admission from those recorded in re-admissions over the subsequent 6 months, providing novel information that can be used in a clinical setting.

Our study has several weaknesses. First, is the lack of detail in HES to establish whether VTE is the cause or consequence of hospitalisation when assessing VTE as baseline. This is a limitation inherent in all hospital-based studies using discharge notes, as primary diagnosis is not necessarily the reason for hospitalisation. Second is the reliability of the diagnostic coding for VTE in HES. This is in terms of sensitivity, as not all VTE events may be recorded in secondary care, as well as specificity, as data to support a VTE diagnosis, such as evidence of anticoagulant treatment, are not available in HES. As we did not have access to outpatient data, and given that the majority of cancer-associated VTE is diagnosed and managed as an outpatient [20], our estimates of the risk of VTE post-discharge are most probably underestimated. Thus, the true burden of VTE in hospitalised cancer patients post-discharge may be greater than we report. However, as VTE events occurring during an inpatient admission would be fully recorded, we do not believe that our rates of VTE during hospitalisation will be underestimated. Third, our study is only able to assess the risk of VTE in people who are hospitalised for cancer, so these results cannot be applied to patients not hospitalised for their cancer (for instance those who die without ever being hospitalised).

Similar to previous studies, we lack information on potential confounders such as stage of disease and comorbidity which have been shown to be associated with risk of VTE. These variables could explain why patients with certain cancer types, and those undergoing chemotherapy, have a higher risk of VTE than others [14, 20]. Finally, as in the case of other studies, it is likely that we have underestimated the number of people receiving chemotherapy as we have only included therapy during hospital admission.

### Comparison with other studies

This current study is consistent with the findings of previous work, that pancreatic cancer is associated with the highest risk of VTE amongst patients hospitalised for cancer [16–18]. With respect to the risk of VTE during hospitalisation, Stein et al. [17] found a similar finding of 2 % risk of VTE in patients hospitalised for cancer between 1979 and 1999. However, a US-based cohort study by Khorana et al. [18] reported a 4.1 % overall risk of VTE during hospitalisation, almost double the risk we report. The risk of VTE may be higher in the USA compared to the UK due to true population differences or different case ascertainment and/or use of prophylaxis. The studies by Levitan et al. [15] and a separate US study specifically including patients with neutropenia (Khorana et al. [16]) both demonstrated that the risk of re-admission for VTE is smaller than during initial hospitalisation, similar to this current study This could be a result of comorbidities, infections, lack of mobility or the effect of various treatments during hospitalisation, which are all associated with risk of VTE or the aforementioned potential for under recording of VTE events occurring post-discharge [21–26].

With respect to the association between age and risk of VTE in cancer patients, there are inconsistent findings in the literature [16, 18, 27]. We have found, in general, that risk of VTE increases with age during initial hospitalisation, apart from cancers with a poor prognosis. The former could be due to increasing baseline risk of VTE with age. The latter finding could be due to older patients with a poor prognosis being more likely to die before having a VTE than younger patients.

Regarding the effect of treatment on the risk of VTE, the study by Khorana et al. [18] is the only previous hospital-based study to examine the association between VTE event and chemotherapy and also found the risk of VTE was higher amongst patients who underwent chemotherapy, than those who did not. However, because the study was not prospective, they were unable to explore the risk of re-admission of VTE, neither were results for chemotherapy stratified by cancer type. In our study, we included episodes of chemotherapy delivered in subsequent day case admissions and as such would have captured this information more comprehensively. This could explain why we found a higher proportion of patients undergoing chemotherapy [18].

To our knowledge, only one study has stratified rates by cancer site and demonstrated how the increase in rates over calendar period was higher in those with a greater rate of VTE [27]. In addition to this, we have demonstrated that the trends in VTE vary not only by cancer site but whether the VTE event occurred during hospitalisation (adjusting for length of stay) or following discharge, with subsequent VTE in patients with cancers of the lung having increased markedly over the 14-year study period.

### Clinical implications

Given that our study and others highlight the varying risk of VTE by cancer site and the higher risk in hospital compared to post-discharge, careful consideration of the patients that would and would not benefit from prophylaxis following hospitalisation is required. For example, young patients with malignant melanoma may experience a net harm from taking in-hospital prophylaxis whereas young patients with pancreatic, lung or liver cancer may benefit. One could argue, however, that for patients with pancreatic cancer, who are at such advanced disease stage and in poor health in general, that prevention of VTE may not be cost effective as they are likely to die short term for other reasons. The relatively low risk of VTE in patients with myeloma could reflect clinicians’ use of routine prophylaxis during chemotherapy as an outpatient and reflects results from other inpatient studies which report VTE rates in myeloma which are similar to the average for all cancer patients) [16, 18].

Our work adds to ongoing research investigating the association of chemotherapy with the development of VTE in patients with cancer. Such an association has been shown in several studies [2, 28–31]. For example, in one population-based case-control study, patients receiving chemotherapy had a higher odds ratio for the development of VTE (6.5) than those not receiving chemotherapy (4.1), when compared with patients without cancer [2]. Our group’s recent work on VTE in breast cancer showed the risk of VTE was tenfold when chemotherapy was treated as a time-varying covariate [31]. Due to limitations of the data in this current study, we have only been able to crudely analyse the effect of chemotherapy on risk of VTE.

Khorana et al. [32] published a risk assessment model to estimate the risk of VTE in patients with cancer receiving chemotherapy (4066 patients) which has set the stage for randomised clinical trials in this area. In this risk model, cancers of the stomach and pancreas were classed as very high risk. Such a classification was supported by data from the sub-group of patients in our study who underwent chemotherapy (which took place an average of 46 days into the 6 month interval), with a high VTE risk (>4 %) also occurring amongst people with oesophageal cancer. Such information could be used to influence the introduction of chemotherapy as a risk factor into some guidelines for specific sub-groups of patients, as has been suggested by the National Comprehensive Cancer Network [33].

We have demonstrated that trends of VTE over time vary considerably by cancer site. For example, in patients with lung cancer, the risk of VTE during hospitalisation doubled between 1998 and 2008 (even after adjusting for length of hospital stay), whereas it fell or only slightly increased for all other cancers. This rise may be explained by greater ascertainment by computerised tomography (CT) scan rather than a real rise. Patients with lung cancer are most likely to get follow-up CT scans than patients with other cancers, and there is increasing CT availability and increasing resolution of scans in the UK.

## Conclusions

This is the first hospital-based study to report the risk of VTE amongst patients with cancer in the UK. When considering clinical guidelines for inpatients, cancer site may need to be taken into account, especially as the risk varies from 0.37 % (malignant melanoma) to 4.89 % (pancreas). There could be more of a focus on early prophylactic use amongst the high-risk cancers immediately following hospitalisation, especially amongst younger patients with pancreatic cancer, and consideration of chemotherapy, as a potential risk factor, in future clinical decision-making may be required.

## Abbreviations

HES, Hospital Episode Statistics; NICE, National Institute for Health and Care Excellence; ONS, Office for National Statistics; VTE, venous thromboembolism

## Additional files

Additional file 1: Figure S1. Risk of first VTE during long stay hospitalisation (≥3 days) by year of cancer diagnosis. (DOCX 20 kb) Additional file 2: Figure S2. Riskfirst VTE during long stay hospitalisation (≥3 days) by year of cancer diagnosis-primary diagnosis only. (DOCX 21 kb) Additional file 3: Figure S3. Rate of VTE six months post-discharge by cancer site. (DOCX 18 kb)
