# Supplementary material for: Risk of venous thromboembolism in hospitalised cancer patients in England—a cohort study
Source: J Hematol Oncol. 2016 Jul 26;9:60. doi: 10.1186/s13045-016-0291-0 (PMC4962547; doi:10.1186/s13045-016-0291-0)
Supplement: Additional file 2: Figure S2. — Riskfirst VTE during long stay hospitalisation (≥3 days) by year of cancer diagnosis-primary diagnosis only. (DOCX 21 kb) [file 13045_2016_291_MOESM2_ESM.docx]

**Supplementary Figure 2**

Error bars=95% confidence intervals
