# Supplementary material for: Risk of venous thromboembolism in hospitalised cancer patients in England—a cohort study
Source: J Hematol Oncol. 2016 Jul 26;9:60. doi: 10.1186/s13045-016-0291-0 (PMC4962547; doi:10.1186/s13045-016-0291-0)
Supplement: Additional file 3: Figure S3. — Rate of VTE six months post-discharge by cancer site. (DOCX 18 kb) [file 13045_2016_291_MOESM3_ESM.docx]

Error bars=95% confidence intervals
